# Unsupervised Machine Learning to Detect and Characterize Barriers to Pre-exposure Prophylaxis Therapy: Multiplatform Social Media Study

**DOI:** 10.2196/35446

**Published:** 2022-04-28

**Authors:** Qing Xu, Matthew C Nali, Tiana McMann, Hector Godinez, Jiawei Li, Yifan He, Mingxiang Cai, Christine Lee, Christine Merenda, Richardae Araojo, Tim Ken Mackey

**Affiliations:** 1 S-3 Research San Diego, CA United States; 2 Global Health Policy and Data Institute San Diego, CA United States; 3 Global Health Program, Department of Anthropology University of California La Jolla, CA United States; 4 Office of Minority Health and Health Equity, U.S. Food and Drug Administration Silver Spring, MD United States

**Keywords:** infoveillance, HIV, minority health, PrEP, social media

## Abstract

**Background:**

Among racial and ethnic minority groups, the risk of HIV infection is an ongoing public health challenge. Pre-exposure prophylaxis (PrEP) is highly effective for preventing HIV when taken as prescribed. However, there is a need to understand the experiences, attitudes, and barriers of PrEP for racial and ethnic minority populations and sexual minority groups.

**Objective:**

This infodemiology study aimed to leverage big data and unsupervised machine learning to identify, characterize, and elucidate experiences and attitudes regarding perceived barriers associated with the uptake and adherence to PrEP therapy. This study also specifically examined shared experiences from racial or ethnic populations and sexual minority groups.

**Methods:**

The study used data mining approaches to collect posts from popular social media platforms such as Twitter, YouTube, Tumblr, Instagram, and Reddit. Posts were selected by filtering for keywords associated with PrEP, HIV, and approved PrEP therapies. We analyzed data using unsupervised machine learning, followed by manual annotation using a deductive coding approach to characterize PrEP and other HIV prevention–related themes discussed by users.

**Results:**

We collected 522,430 posts over a 60-day period, including 408,637 (78.22%) tweets, 13,768 (2.63%) YouTube comments, 8728 (1.67%) Tumblr posts, 88,177 (16.88%) Instagram posts, and 3120 (0.6%) Reddit posts. After applying unsupervised machine learning and content analysis, 785 posts were identified that specifically related to barriers to PrEP, and they were grouped into three major thematic domains: provider level (13/785, 1.7%), patient level (570/785, 72.6%), and community level (166/785, 21.1%). The main barriers identified in these categories included those associated with knowledge (lack of knowledge about PrEP), access issues (lack of insurance coverage, no prescription, and impact of COVID-19 pandemic), and adherence (subjective reasons for why users terminated PrEP or decided not to start PrEP, such as side effects, alternative HIV prevention measures, and social stigma). Among the 785 PrEP posts, we identified 320 (40.8%) posts where users self-identified as racial or ethnic minority or as a sexual minority group with their specific PrEP barriers and concerns.

**Conclusions:**

Both objective and subjective reasons were identified as barriers reported by social media users when initiating, accessing, and adhering to PrEP. Though ample evidence supports PrEP as an effective HIV prevention strategy, user-generated posts nevertheless provide insights into what barriers are preventing people from broader adoption of PrEP, including topics that are specific to 2 different groups of sexual minority groups and racial and ethnic minority populations. Results have the potential to inform future health promotion and regulatory science approaches that can reach these HIV and AIDS communities that may benefit from PrEP.

## Introduction

### Pre-exposure Prophylaxis Use Among Minority Populations

HIV remains one of the world’s most pressing global public health challenges. According to the Joint United Nations Programme on HIV and AIDS, there were approximately 38 million people across the world living with HIV in 2019 [1], and in the same year, an estimated 1.7 million people became newly infected with HIV [2]. Concomitantly, only about 24.5 million people had access to antiretroviral therapy, including pre-exposure prophylaxis (PrEP), a proven and safe method to prevent HIV transmission [2-6]. For example, in the United States, only approximately 7% of people who meet the indication for use of PrEP are prescribed PrEP and adhere to protocols [7,8]. These numbers fall well short of the ambitious *90-90-90* targets set by Joint United Nations Programme on HIV and AIDS to have 90% of HIV-infected individuals diagnosed, receiving antiretroviral therapy, and achieving viral suppression, which is also impacted by challenges associated with adherence to treatment [6,8]. Among those at risk for HIV, certain racial and ethnic minorities remain disproportionately impacted and may face structural and economic barriers associated with the access and ability to start HIV prevention and treatment services such as PrEP [9-11].

For example, according to data from the US Center for Disease Control and Prevention, Blacks or African Americans and Hispanics or Latinos comprised 41% and 23% of people living with HIV, respectively, and Black or African American and Hispanic or Latino men who have sex with men accounted for 26% and 22% of new HIV infections in 2018, respectively [12]. In addition, Black or African American women remain at higher risk for HIV transmission than White and Hispanic or Latina women, and African American or Black and Hispanic or Latina women’s PrEP uptake lags behind that of White women [13,14]. Hence, even though PrEP is a highly effective HIV prevention modality, its adoption has not yet become the standard of care among certain racial and ethnic minority populations and sexual minority groups who are at heightened risk for HIV [15,16].

Clinical studies have demonstrated the effectiveness of PrEP in preventing HIV when taken as prescribed, with data from the Center for Disease Control and Prevention finding that it reduces HIV transmission from sex by approximately 99% and by at least 74% for people who inject drugs [17,18]. However, self-perceived and objective barriers continue to hinder PrEP’s widespread use [13]. For example, barriers across the PrEP continuum of care in an integrated health care setting were more pronounced for racial and ethnic minority patients, individuals with lower socioeconomic status, and those with substance use disorder, with PrEP attrition associated with HIV infection [19].

### Social Media Platforms, HIV, and Infodemiology

To further encourage PrEP uptake, social media platforms have increasingly evolved into spaces to deliver health information and for users to actively report and discuss their health behavior, including in the context of HIV [20-22]. Owing to the ability of these platforms to share information and reach diverse audiences, health communication and promotion efforts aimed at increasing awareness about PrEP and destigmatizing its use are a possibility [1,23]. Certain platforms, such as Instagram and Twitter, are also popular among Black or African American and Hispanic or Latino youth, highlighting the potential for social media to generate better understanding into the knowledge, attitudes, and behaviors of specific minority groups for health topics [1]. Leveraging publicly available social media data using *infodemiology* approaches (ie, the science of distribution and determinants of information in an electronic medium, with the aim of informing public health), this study analyzed user-generated conversations about PrEP from a multiplatform perspective, including examining the experiences of racial and ethnic groups and sexual minorities [24].

## Methods

### Ethics Approval

This study has been approved by WCG IRB. WCG IRB is registered with the Office for Human Research Protections and US Food and Drug Administration (FDA) as IRB00000533.

### Data Collection

**Figure 1:**
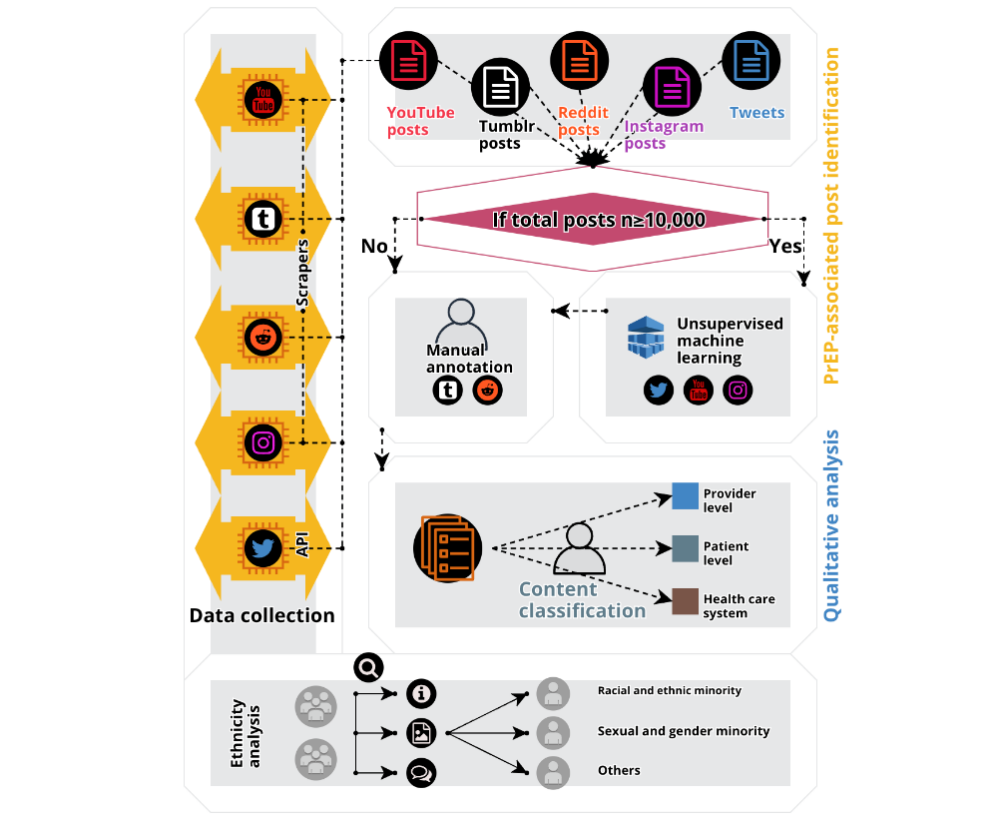
Methodology summary and flowchart. API: application programming interface; PrEP: pre-exposure prophylaxis.

We first generated a list of PrEP- and HIV-associated keywords and hashtags by manually searching posts on social media platforms that were selected for this study. These included a baseline set of general terms associated with HIV, PrEP, and FDA-approved PrEP medications. We searched this initial set of keywords on Twitter, Tumblr, Reddit, YouTube, and Instagram, which enabled us to collect additional hashtags and keywords associated with HIV prevention, HIV treatment, and HIV disease experiences, which also included concurrent user discussions about PrEP therapy as observed in results from the first 100 returned posts for each searched term. This enabled us to generate a more comprehensive list of associated keywords and hashtags specific to social media conversations related to HIV prevention and PrEP, which were then further used for a broader and structured data collection approach on the 5 study platforms selected (refer to Table 1 for the full list of study keywords and hashtags). We chose these platforms based on their general popularity, accessibility of publicly available data, and diversity in methods of web-based and social communication and interaction (eg, microblogging sites [Twitter and Tumblr], a news aggregation and discussion site [Reddit], a video sharing site [YouTube], and a photo and video social networking site [Instagram]). We also decided to pursue a multiplatform infodemiology study on the basis of seeking a variety of user discussions from different and diverse web-based communities (eg, social media platforms chosen for this study have different user demographics and audiences), whereas a single platform study may have yielded less diversity of users, topics, and themes related to PrEP. We used the public streaming Twitter application programming interface to collect tweets on Twitter and an automated web scraper developed in the programming language Python using the Beautiful Soup package to collect publicly available posts from Tumblr, YouTube, Reddit, and Instagram. Posts were collected from all 5 platforms simultaneously over a 60-day study period (from October 13, 2020, to December 11, 2020) and contained both retrospective data (eg, posts that occurred before the date of collection) and prospective data (eg, Twitter posts were collected starting on the date of querying the application programming interface). A visual summary of the study methodology used is provided in Figure 1.

**Table 1:** Selected pre-exposure prophylaxis (PrEP)- and HIV-related keywords and hashtags.

	Additional keywords	Hashtags
PrEP-related keywords or hashtags	*PrEP; Post-exposure prophylaxis*	#Iwantprepnow; #PrepworkPepfarsaveslives; #prep4blackqueermen; #ondemandprep
HIV-related keywords or hashtags	*HIV Clinic; Unsafe sex; POZ; ART; Serosort; The disease; The ick*	#HIVawareness; #HIVprevention; #Queerhealth; #knowyourstatus; #Imstoppinghiv#undetectableequalsuntransmittable; #hivundetectable; #hivpoz; #hivprevention; #uequalu; #uequalsu
Medication-related keywords or hashtags	*Descovy; Truvada; Tenemine; Tivicay; Aluvia; AIDS cocktail; Meds; Cabotegravir; Ceftriaxone; Doxycycline; Tenvir; Tenofovir; Duomune; Emtricitabine*	#Truvada; #showyourpill; #Truvadawhore; #Chuvadetrovada; #hivmeds; #truvadaforprep; #preppill

### Unsupervised Machine Learning

To identify, characterize, and elucidate the experiences, attitudes, and perceived barriers associated with the adoption and adherence to PrEP therapy, we used a form of unsupervised machine learning in the family of topic modeling and natural language processing to identify topics and word groupings relevant to the study objectives. We used the biterm topic model (BTM), which is an unsupervised machine learning approach designed to detect patterns in the data and summarize the entire corpus of text into distinct highly correlated categories [25-30]. BTM can be used to sort short text into highly prevalent themes without the need of predetermined training data and has been previously used for the exploration of other public health topics [25-30]. Groups of social media messages or text containing the same word-related themes are categorized into clusters, and the main themes of those clusters are considered as the topic of the text aggregation, which is then split into a bag of words where a discrete probability distribution for each theme is generated [31].

Using BTM, we identified topic clusters with word groupings, frequencies, and characteristics that appeared to be related to user conversations associated with HIV prevention or PrEP (*signal clusters*) and then extracted social media posts from these topic clusters for manual annotation. For example, signal clusters that contained a high frequency of PrEP-specific keywords for the outputted topic and also included verb word groupings more indicative of user-generated syntax were prioritized and labeled as potential signal clusters. We set a total number of k=20 different clusters (ie, total number of topics for BTM to output), resulting in texts with similar themes put into the same clusters. To find the appropriate k value, we used a topic coherence score to determine the k value. Coherence score is used to measure the performance of a topic model with different number of clusters and can help distinguish between topics that are semantically interpretable and topics that are artifacts of statistical inference. We test 5 different k values (k=10, 20, 30, 40, and 50) for each data set and found that when k=20, we generated the highest coherence score, and this score did not change significantly with an increase in the k value. On the basis of the results generated from BTM, associated social media posts highly correlated with signal clusters were then extracted and reviewed using manual annotation.

Posts were deemed as *signal* posts (ie, relevant social media posts to the study aims) if they were (1) user generated (ie, not posted by organizations or media outlets) and (2) discussed a topic relevant to PrEP therapy access, use, adherence, and associated barriers. Posts related solely to news or media coverage about PrEP, advertisements of PrEP services or treatment, and posts not related to PrEP (eg, such as the use of *prep* for the description of general food preparation) were excluded from further analysis. In addition, based on the number of posts collected from each platform, we used a protocol of using either (1) BTM in combination with manual annotation of posts for platforms where there were greater than 10,000 posts or (2) solely relying on manual annotation of posts for platforms with less than 10,000 total posts. The 10,000-post cutoff was deemed appropriate given previous studies that have relied on manual annotation for similarly sized social media data sets and the relative imprecision of BTM compared with manual annotation when examining smaller sized data sets [32].

### Content Analysis

To classify the content of posts identified as potential *signal* posts following BTM and manual annotation, we used a deductive coding approach based on the socioecological perspective outline (SEPO), which focuses on barriers to PrEP [33-36]. All posts were first reviewed by the first author (QX), and notes were taken on general themes of posts from which an initial code list was created. Following the SEPO [36,37], all detected themes were deductively classified in 3 intervention levels: *Individual and Relationships Domains: Provider Level*, *Individual and Relationships Domains:* *Patient Level,* and *Community Domains: Health care System Level* (refer to Table 2 for description). Reported categories of barriers to PrEP and other forms of HIV prevention were adopted from SEPO and new subcodes adopted throughout our process of content coding. Subcodes that were not covered under SEPO were inductively added to the codebook under the 3 parent codes based on the conceptual domain and intervention level of the new theme.

To further elucidate potential patient decision-making rationale about the use of PrEP, we also conducted an additional round of deductive coding by adopting the consumer decision-making process model (CDMPM). For CDMPM, we assessed the potential impact on PrEP access and barriers to access by categorizing all signal posts into the 5 stages of the CDMPM decision process (Table 2) [38]. The categorization and inclusion criteria for each of the five stages are as follows: (1) need recognition—post shows the users recognizing their risk of contracting HIV, but have not started looking for a protection method, (2) information search—post shows users have recognized there is a risk of HIV and are looking for information on protection and prevention, (3) evaluation of alternatives—posts comparing the use of PrEP and other alternative prevention methods (eg, condoms), (4) purchase (using)—post reflects users with the intent of using PrEP or have started using PrEP and posts that also show users deciding not to use PrEP, and (5) postusing behavior—posts discussing themes after PrEP use has been initiated (eg, adherence and conversely terminating the use of PrEP for different reasons).

First (QX) and second (MCN) authors coded all posts independently and achieved high intercoder reliability for post signal coding (Cohen *κ*=93.46). A final coded data set was reviewed by the third (TM) and fourth (HG) authors to assess if any differences in code definitions and application occurred. First through fourth authors reconciled differences and reached consensus on the correct classification.

**Table 2:** Description of socioecological perspective outline (SEPO) and consumer decision-making process model (CDMPM).

Model type and levels or stages		Description
**SEPO**		
	Individual and relationships domains: provider level	Focused on primary care physicians, HIV and infectious disease specialists, pharmacists, and nurse practitioners
	Individual and relationships domains: patient level	PrEP^a^ patients’ and potential patients’ attitudes, beliefs, and experiences
	Community and system domains: health care system level	System-level barriers to PrEP implementation
**CDMPM**		
	Need recognition	Recognition of risk for contracting HIV
	Information search	Looking for information on HIV protection and prevention
	Evaluation of alternatives	Comparing the use of PrEP and other alternative prevention methods
	Purchase (using)	Intent of using PrEP or have started using PrEP or deciding not to use PrEP
	Postpurchase behavior (postusing behavior)	Discussing themes after PrEP use (satisfied or dissatisfied)

^a^PrEP: pre-exposure prophylaxis.

### User Metadata Analysis

To further characterize the potential challenges associated with PrEP uptake, access, and adherence specific to minority populations, we also examined publicly available metadata of users associated with signal posts for any potential identifiable minority status. In this study, minority groups included racial and ethnic minorities as well as sexual and gender minorities. This included 5 major racial and ethnic groups: Blacks or African Americans, American Indians and Alaska Natives, Asians, Native Hawaiian or other Pacific Islanders, and Hispanics or Latinos. It also included a broad classification of 5 sexual and gender minorities: lesbian, gay, bisexual, transgender, and queer users. The classification used only publicly available profile data and information from the last 10 posts from the user’s account or timeline to assess whether there was sufficient information to identify at least one of the above-mentioned minority classes. These data were collected for purposes of aggregation, and no results contained in this study include individually identifiable information or make any representation to the accuracy of a claimed minority classification of a user.

## Results

### Overview

We collected 522,420 posts over the 60-day study period among all collected retrospective and prospective posts across the 5 social media platforms in this study. Breakdown per platform for the 522,420 posts is as follows: 408,637 (78.22%) tweets, 13,768 (2.63%) YouTube comments, 8728 (1.67%) Tumblr posts, 88,177 (16.71%) Instagram posts, and 3120 (0.6%) Reddit posts. After applying our approach of BTM and manual annotation to confirm signal posts, 785 posts were identified as associated with PrEP-related topics, which comprised posts from 715 unique social media user accounts. The 785 signal posts were identified from Twitter (n=430, 54.8%), Reddit (n=256, 32.6%), Instagram (n=41, 5.2%), Tumblr (n=41, 5.2%), and YouTube (n=17, 2.2%; Table 3). The period covered by this subset of signal posts was from June 4, 2015 (earliest posted on Instagram), to November 23, 2020 (latest posted on Twitter). According to the time duration of signal posts for specific platforms, Instagram had the longest period of signal posts detected (June 4, 2015, to October 30, 2020), and Twitter had the shortest period of coverage (October 14, 2020, to November 23, 2020) because of the prospective nature of the data collection process. Generally, these periods coincide with very early retrospective dates around the time of PrEP therapy introduction (eg, US FDA approval) and more recent conversations about PrEP closer to the study data collection period.

**Table 3:** Volume and period of coverage of the collected posts and signal posts.

Platforms	Collected posts						Signal posts				
	Total number	Period of posts	Identified users	Day posts	Day posts, mean (SD)	Total number		Period of posts	Identified users	Day posts	Day posts, mean (SD)
Instagram	88,177	April 4, 2012, to October 29, 2020.	23,654	28.36	8 (98.76)	41		June 4, 2015, to October 30, 2020.	37	0.021	0 (0.15)
Reddit	3120	February 28, 2007, to November 25, 2020.	2772	0.62	0 (3.17)	256		January 22, 2016, to November 10, 2020.	250	0.15	0 (0.54)
Tumblr	8728	August 16, 2010, to November 18, 2020.	5714	2.33	0 (7.29)	41		August 22, 2015, to June 17, 2020.	31	0.023	0 (0.19)
Twitter	408,637	October 13, 2020, to November 25, 2020.	207,368	9503.19	15,537 (6078.02)	430		October 14, 2020, to November 23, 2020.	383	10.75	2 (29.53)
YouTube	13,758	May 5, 2010, to November 16, 2020.	12,185	3.58	0 (2.09)	17		July 26, 2019, to November 1, 2020.	14	0.037	0 (0.16)

### Content Analysis: SEPO

On the basis of our qualitative analysis and deductive coding approach, 39 topics based on the SEPO and CDMPM were derived. A complete breakdown of the stratification of these codes and subcodes for each social media platform is provided in Multimedia Appendix 1. Following the SEPO framework [37], all detected topics were first classified into the 3 parent domains: provider level (13/785, 1.7% posts), patient level (570/785, 72.6% posts), and community level (166/785, 21.2% posts; refer to Table 4 for codes and subcodes and deidentified examples). From the posts identified in the provider domain, which focuses on barriers perceived by users to occur at the health care provider level, discussions focused on themes related to providers’ knowledge or lack thereof about PrEP (7/13, 54% posts; A-1-a, A-1-b), provider’s attitudes toward PrEP and patients seeking care (5/13, 38% posts; A-2-a), and an instance where a provider unintentionally failed to renew a PrEP prescription (1/13, 8% posts; A-3-a). For example, a group of conversations in this level focused on how primary physicians could not prescribe PrEP because they did not know the correct application or use of the medication.

In the patient domain, which focuses on barriers that are perceived to originate from a patient or prospective patient’s attitudes, knowledge, and behavior, the highest number of themes detected related to the SEPO knowledge topic (528/570, 92.6% posts; B-1-[a-h]), which included patients having low awareness of PrEP, lack of knowledge about approved PrEP medications, issues with insurance coverage, and lack of local health resources to access PrEP. In addition, patients also mentioned barriers regarding their attitudes and beliefs shaped by their experiences (157/570, 27.5% posts; B-2-[a-l]), which included subtopics reporting users’ apprehension about side effects associated with PrEP, prioritizing another personal health issue over HIV prevention, concerns about contraindication of PrEP with other illicit drugs (eg, recreational drug use), distrust of the medical system, users’ self-evaluation as being at low risk for HIV and foregoing PrEP, and a preference to use other HIV prevention measure (eg, using a condom during sexual intercourse). We also observed that patients’ experiences related to knowledge and barriers to PrEP therapy varied greatly, such as misinformation on COVID-19 (eg, users stating that they were protected or immune from COVID-19 if they were on PrEP therapy) and use of PrEP that were coded as expressions of adherence, expressions of interest to donate unused PrEP to others, concerns about contraindication with other illicit drug use, and more common experiences of users reporting terminating PrEP because they were experiencing adverse reactions.

The final SEPO level focused on PrEP barriers originating from more structured community-related topics reported by users. The themes primarily focused on issues associated with communication and lack of awareness about PrEP (5/166, 3% posts; C-1-a), general lack of government funding for PrEP programs and its impact on insurance coverage (90/166, 54.2% posts; C-2-a and C-2-b), barriers related to health care referral system, issues of inadequate transportation to clinics (C-3-[a-i]), challenges with prescription filling (48/166, 28.9% posts), and other barriers related to PrEP medication characteristics (eg, route of administration and daily dosing schedule; 21/166, 12.7% posts; C-4-a). Other socially contextual issues were also detected such as the perception of population-specific barriers and stigma to PrEP (54/166, 32.5% posts; C-5-[a-e]), discussion about the need for HIV testing and effectiveness of PrEP therapy for injection drug users, and a perceived lack of inclusivity for clinical trials associated with PrEP therapy for certain sexual minority groups. Finally, community concerns included users reporting similar concerns to those observed at the patient level regarding loss of coverage for PrEP patients because of changes in insurance policies or limited access to HIV clinical services because of the COVID-19 pandemic.

**Table 4:** Top 3 topics in 4 ethnic minority groups.

Ethnic minority groups and top 3 topic code numbers				Topic description		Number of posts (N=162), n (%)		Sexual and gender minorities (N=19), n (%)
**Blacks or African Americans (n=129)**								11 (57.8)
	B-1-e		Comparing different drugs (Truvada and Descovy)		39 (24.1)			
	B-1-b		Sharing PrEP^a^ knowledge or experience with other patients		33 (20.4)			
	B-1-d		Asking about knowledge related to the use, effectiveness, or side effects of PrEP		10 (6.2)			
**Asians (n=19)**								3 (15.8)
	B-1-b		Sharing PrEP knowledge or experience with other patients		11 (6.8)			
	B-1-e		Comparing different drugs (Truvada and Descovy)		3 (1.9)			
	B-2-k		Self-evaluated as low risk		2 (1.2)			
**Hispanics (n=12)**								5 (26.3)
	B-1-e		Comparing different drugs (Truvada and Descovy)		5 (3.1)			
	B-1-b		Sharing PrEP knowledge or experience with other patients		3 (1.9)			
	C-5-b		Lack of transinclusive marketing of PrEP		2 (1.2)			
**American Indians and Alaskan Natives (n=2)**								0 (0)
	B-1-e		Comparing different drugs (Truvada and Descovy)		1 (0.6)			
	B-2-a		Side effects, effectiveness, toxicities, and interaction with feminizing hormones		1 (0.6)			
Total (n=162)						110 (67.9)		19 (100)

^a^PrEP: pre-exposure prophylaxis.

### Content Analysis: CDMPM

In addition to coding user-generated data for SEPO domains, we also assessed the stages of the patient PrEP decision-making processes per the CDMPM. We first removed all signal posts not associated with the patient decision-making process based on our first deductive coding round of SEPO themes and recoded the data for CDMPM categorized stages (eg, posts that discussed HIV and AIDS prevention technology and certain information-seeking categories were removed). Similar to themes generated in the SEPO domains, we observed that users discussed overlapping concerns throughout the patient decision-making process, including information-seeking behavior and how it impacted decisions to seek PrEP therapy (information search stage), users’ decisions to use or not use PrEP (purchase or use stage), and what factors led to continued adherence or termination of PrEP therapy (postpurchase behavior level).

**Figure 2:**
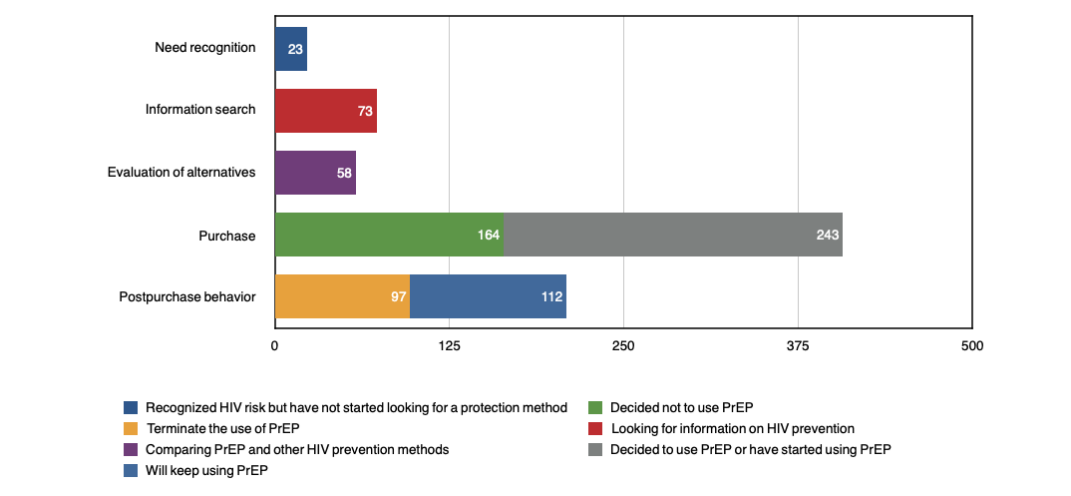
Number of posts at different stages of the patient decision process. PrEP: pre-exposure prophylaxis.

Specifically, within this subset of patient-focused data, the most prominent user conversations were found in the CDMPM purchase or use stage for PrEP (407/770, 52.9% posts; Figure 2). Specifically, of the 407 posts, we detected 243 (59.7%) posts where users reflected on their decision to use PrEP or had already started using PrEP and 164 (40.3%) posts where users expressed their intent to not use PrEP. Primarily, patients stated that their decision to ultimately use PrEP was based on their research on the overall effectiveness of its ability to prevent HIV or that they relied on a physician’s recommendation about seeking therapy, particularly when the patient knew they were going to engage in high-risk behavior. In contrast, users’ common rationale for not using PrEP included concerns about unwanted side effects (also because of observations from other users who reported experiencing adverse side effects), inability to access because of lack of insurance coverage, users believing they were not engaged in high-risk sexual behavior, and users comparing the overall effectiveness of PrEP with condoms (eg, opinions that condoms had greater utility as they prevent both HIV and sexually transmitted diseases or sexually transmitted infections).

The second most prominent CDMPM stage detected from signal posts was at the postpurchase behavior level (209/770, 27.1% posts), which included 14.5% (112/770) posts describing continued use of and adherence to PrEP after the initiation of treatment. Conversely, 12.6% (97/770) of the posts included conversations detailing users reporting termination of PrEP for reasons such as financial issues and lack of affordability, lack of access to HIV and AIDS clinics and other related medical resources, and barriers to continuing PrEP because of other health issues experienced by patients. Health issues experienced by patients that led to PrEP discontinuance included side effects from PrEP therapy (eg, unwanted weight gain, headache, nausea, and loss of appetite), the need and complexity to manage multiple health concerns other than HIV and AIDS, and the presence of other health conditions that interfered with PrEP therapy.

Finally, 9.5% (73/770) posts were categorized as occurring in the CDMPM information search stage and included discussions about information-seeking behavior, where users recognized or became concerned about potential HIV risk and then sought more information on how to protect themselves, including inquiring about PrEP. A total of 7.5% (58/770) of these posts included discussions comparing the use of PrEP with condom use, and 2.9% (23/770) posts were categorized as in the need recognition stage, where users recognized their risk but did not mention any specific measure of protection or prevention action subsequently taken.

### User Metadata Analysis

From 785 signal posts confirmed as associated with PrEP, 320 (44.8%) posts had sufficient metadata in publicly available profile or post information to allow for identification of at least one racial or ethnic minority group (127/320, 39.7% posts) or at least one sexual or gender minority group (233/320, 72.8% posts). Among all racial and ethnic minority groups and sexual minority group–related accounts, sexual and gender minorities were the largest group of users (n=233). Their posts covered 32 PrEP-coded topics, with the top topic inquiring about information related to the use, effectiveness, or side effects of PrEP (B-1-d). The second top topic discussed side effects, effectiveness, toxicities, and interaction with feminizing hormones (B-2-a), and the third top topic was associated with users sharing PrEP knowledge or experience with other users (B-1-b).

The largest volume of racial and ethnic user posts (total n=129) were self-identified as Blacks or African Americans (96/129, 74.4%), followed by a much smaller volume of Asians (19/129, 14.7%), Hispanics or Latinos (12/129, 9.3%), and American Indians and Alaskan Natives (2/129, 1.6%). According to our analysis, we did not detect any users self-identified as Native Hawaiian or Other Pacific Islanders. On the basis of a review of codes and subcodes for these racial and ethnic minority user-specific posts, we identified 11 PrEP conversation topics most prevalent among Black or African American users, with the top topic associated with users comparing PrEP-approved treatment options (Table 4; code number B-1-e), the second associated with users discussing their experiences and knowledge about PrEP with their peers (B-1-b), and the third topic including general inquiries about information related to PrEP use. Users self-identified as Asian covered 8 topics, with the top topic related to sharing PrEP knowledge or experiences with other users (B-1-b), the second topic comparing PrEP treatment options (B-1-e), and the third topic discussing how users self-evaluate whether they are at low risk of contracting HIV and AIDS (B-2-k). For users self-identifying as Hispanics or Latinos, 7 topics were detected, with the top topic again comprising discussions comparing PrEP treatment options (B-1-e), the second discussing general knowledge and experiences with PrEP (B-1-b), and the third where users felt there was a lack of transinclusive marketing of PrEP but did not explicitly claim transgender affiliation (C-5-b). Owing to the low volume of users detected as American Indian and Alaskan Native, only 2 topics were detected for this group: comparing PrEP treatment options (B-1-e) and a topic related to concerns about PrEP side effects, effectiveness, toxicities, and interaction with feminizing hormones (B-2-a).

## Discussion

### Principal Findings

In this multiplatform infodemiology study, we analyzed just over a half million social media posts from popular platforms such as Twitter, YouTube, Instagram, Reddit, and Tumblr using a combination of unsupervised machine learning and manual annotation. This resulted in 785 user-generated posts that included conversations about PrEP and other HIV prevention–related topics confirmed through manual annotation and deductively coded for themes associated with the SEPO specific to PrEP barriers and the CDMPM adapted for PrEP patient decision-making stages. Many of these signal posts (320/785, 40.8%) were identified as a racial or ethnic minority population or sexual minority groups. Most users (233/785, 29.7%) were sexual or gender minority status, followed by Blacks or African Americans.

Of all the SEPO levels reviewed, the patient level had more than three-quarters of the volume of all PrEP-related social media conversations. This indicates that users who belong to the HIV and AIDS community or who may be at higher risk of HIV transmission often associate barriers to PrEP therapy, as influenced by patient knowledge, attitudes, and behaviors toward PrEP, though barriers at the provider and community levels were also detected. The largest volume of data for our CDMPM analysis focused on the purchase or use stage of the PrEP decision-making process, indicating that users actively discuss their intent to use or not use PrEP on social media, which may also be influenced by exposure to different forms of information and overall knowledge, or lack thereof, about the benefits of PrEP therapy.

Importantly, regardless of the platform used or coding framework applied, overlapping topics related to specific barriers experienced by users that may impede PrEP therapy were detected, indicating that these challenges may represent possible priority areas for the future design of HIV prevention interventions and education aimed at promoting PrEP use. In fact, the theme with the largest total volume of posts centered around knowledge about PrEP, including lack of knowledge about already-approved PrEP medications; whether treatment was covered by insurance; and overall user perception regarding the inadequacy of resources, communication, and awareness about PrEP, all occurring at multiple SEPO levels and throughout the CDMPM decision-making processes. For example, users noted that providers and patients both lacked knowledge about the benefits of PrEP therapy.

Beyond knowledge-related topics, users also reported structural barriers to accessing PrEP therapy at different SEPO and CDMPM levels or stages. For example, some users reported that providers lacked sufficient knowledge about PrEP and failed to prescribe it even when it was beneficial for a patient or appropriately indicated for a patient’s level of HIV risk. Other examples of barriers included medication insurance coverage issues that impacted access and affordability of PrEP, other external financial and health challenges among these patients, the inability to access HIV and AIDS clinics (also because of disruptions occurring from the COVID-19 pandemic), failure to receive a referral for HIV prevention treatment, and lack of transportation to clinics. This was coupled with different perceptions about perceived risks associated with PrEP therapy that impacted both uptake and adherence, including several posts of users expressing concerns about side effects and other users openly discussing their adverse health experiences. Other reported attitudes and experiences that could further exacerbate these structural barriers included users harboring distrust in the medical system, believing they were at low risk of HIV, and concerns about stigma associated with HIV and PrEP.

Hence, the results of this study, although primarily exploratory, provide additional insights into the specific barriers experienced across the HIV and AIDS PrEP care continuum, as expressed by a diverse audience of social media users. In fact, it appears that many users who identified as sexual minorities are primarily concerned about the effectiveness and potential side effects associated with different PrEP therapies. Sexual minority users were also concerned about issues regarding equitable and diverse representation in clinical trials and marketing of PrEP, topics relevant given that this group is disproportionately impacted by HIV and AIDS [39]. For users associating with select racial and ethnic minority groups, many users were uncertain about the differences between the approved PrEP treatment options, and we also observed that these unique patient communities actively discuss PrEP knowledge and experiences among their digital peers. In addition, African Americans or Blacks, a minority group that accounts for a higher proportion of new HIV diagnoses and those living with HIV compared with other ethnic groups, made up the highest volume of minority web-based users identified in this study, highlighting the disproportionate impact that HIV has on both offline and web-based communities.

Finally, examining the breakdown of PrEP user conversations by specific social media platforms, we found that Twitter yielded the largest absolute volume of signal data, but the signal to noise ratio (ie, the number of posts relevant to PrEP vs nonrelevant content) was relatively high. In contrast, our analysis of Reddit data found that it included a high volume of signal relative to the smaller amount of data collected and had the most diversity of coded topics (including 252 total signal posts that comprised 102 posts under the patient level, 71 at the community level, and 9 at the provider level). The high volume of codes in Reddit posts is also attributable to the long text nature of posts and subreddits reviewed, which can include multiple codes in 1 post. Reddit also had the highest volume of posts associated with users identified as a sexual minority. A full breakdown of the top 3 topics detected on each of the different social media platforms is available in Multimedia Appendix 2. Overall, these results indicate that different groups of users may have distinct and different PrEP-related conversations that occur on platforms that represent different and distinct web-based communities, all of which form a unique communication and peer-to-peer environment discussing relevant HIV and AIDS topics, including PrEP.

Results from this study have the potential to inform and generate additional research questions that examine how social media users discuss their experiences, attitudes, and behaviors regarding PrEP, with an aim of increasing the understanding of diverse voices and perspectives [40,41]. Importantly, the popularity, ubiquity, and relative anonymity of these platforms may represent an important data source to investigate sensitive health topics such as HIV and PrEP, particularly as users become more comfortable with discussing these issues in web-based patient-centric communities. This study additionally adds information to inform risk communication and health literacy objectives by better understanding existing knowledge gaps about PrEP and how to target health communication and promotion to specific web-based, diverse, and at-risk populations that may also engage in high-risk behaviors [42,43]. Both of these are important considerations for regulatory decision makers trying to amplify the patient voice and global goals to provide HIV treatment to all those who need it [40].

### Limitations

This study has certain limitations. First, we only collected data from 5 selected social media platforms and limited study keywords to English. This likely biased the study results to native English speakers, excluding minorities for whom English is a second language or those who do not speak English. Hence, the findings are not generalizable to all PrEP social media conversations occurring among web-based users. Our PrEP- and HIV-related keywords and filtered terms were also chosen based on our manual searches conducted directly on the platforms, but they may not have been inclusive of all PrEP-related keywords or other HIV drugs or treatments (eg, bictegravir, emtricitabine, tenofovir alafenamide, abacavir, dolutegravir, and lamivudine) that may nevertheless generate PrEP-related conversations or discussions about HIV prevention behavior. More specifically, this study did not intend to create a comprehensive list of HIV treatment keywords that might occur alongside PrEP-related conversations. Hence, future studies should expand data collection and analysis approaches to different phrases associated with an individuals’ HIV-related risk behavior; for example, sharing needles and unsafe or unprotected sex, to obtain a more representative corpus of social media conversations. In addition, among all collected data, we observed imbalanced data sets, specifically the oversampling of collected tweets that could result in bias during the data analysis and content coding phase. To address data set imbalances in multiplatform infodemiology studies, future studies should adopt controls, such as the Synthetic Minority Oversampling Technique, to mitigate potential bias or normalize data in ways that make them more representative of the number of users or imbalance of users on each platform. We also did not cross-validate the veracity of users’ race and ethnicity. We determined users’ race and ethnicity based on the users’ publicly available metadata, public posts, and profile image. Hence, our classification of user’s race and ethnicity could be inaccurate because of poor quality data or inaccurate user reporting. Future studies should explore combining multiple data layers from different sources to better validate user’s race and ethnicity or use more traditional approaches of data collection (eg, survey instruments and focus groups). This study also did not focus on specific thematic detection of misinformation or inaccurate information regarding PrEP therapy, though these conversations were observed in our data set. Future studies should consider focusing on detection and characterization of specific misinformation or incorrect information that may impact user HIV prevention seeking or HIV prevention behavior, as it specifically occurs on social media platforms. Finally, because of the exploratory nature of this study and the lack of available training data related to PrEP behavior and information seeking, we relied on the use of unsupervised topic modeling using BTM to categorize texts into different groups and selected groups that had keywords relevant to PrEP-related conversations. However, this approach may preclude signal text with a small volume that may be obscured by nonsignal text with higher volume during the topic modeling output phase. To address this limitation, future studies should explore using pretrained natural language processing models (eg, Bidirectional Encoder Representations from Transformers or other forms of supervised machine learning approaches when sufficient training data are available.

### Conclusions

The results from this multiplatform infodemiology study provide additional insights into the challenges faced by diverse web-based patient populations when seeking PrEP therapy. It appears that existing barriers are influenced by a multitude of factors, either they be subjectively based on a user’s experiences or more structural to the HIV risk environment and challenges associated with the HIV care continuum. Future research should continue to assess the utility of data derived from social media platforms to help better understand the real-world barriers to PrEP experienced at different intervention levels and in the patient decision-making process, with the ultimate goal of improving uptake and adherence to this critical tool needed to reduce the burden of HIV.

## Appendix

Multimedia Appendix 1 Code list and identified topic themes.

Multimedia Appendix 2 Breakdown of topics by platform.

## Abbreviations

BTM: biterm topic model
CDMPM: consumer decision-making process model
FDA: Food and Drug Administration
HHS: Health and Human Services
PrEP: pre-exposure prophylaxis
SEPO: socioecological perspective outline
