# Supplementary material for: Unsupervised Machine Learning to Detect and Characterize Barriers to Pre-exposure Prophylaxis Therapy: Multiplatform Social Media Study
Source: JMIR Infodemiology. 2022 Apr 28;2(1):e35446. doi: 10.2196/35446 (PMC10014091; doi:10.2196/35446)
Supplement: Multimedia Appendix 1 [file infodemiology_v2i1e35446_app1.docx]

**Multimedia Appendix 1**

Supplementary Table 1: Code list and identified topic themes (including paraphrased examples).

| Code name and code number | | | Description | Examples (paraphrased) | Number of posts (N=785), n (%) |
| --- | --- | --- | --- | --- | --- |
| **Provider level (A)** | | | | | 13 (1.66) |
|  | **Knowledge (A-1)** | | | | |
|  |  | (A-1-a) | Lack of training in PrEP^a^ provision | “My 30-year-old primary, who I may mention is new out of residency, had never heard of people taking PrEP for pre-exposure. PrEP isn’t exactly cutting-edge medicine anymore...”—Tumblr | 6 (46%) |
|  |  | (A-1-b) | Disagreement or uncertainty about appropriate PrEP patients | “I live in a small city in Texas, and I went to my GP to try and receive Truvada. I knew I was going to be taking a test and then get a Rx script written, but that isn’t what happened. My GP said they don’t prescribe Truvada and only the CDC does this, which is a lie. They didn’t perform tests on me and still charge me”—Reddit | 1 (8%) |
|  | Attitudes and beliefs (A-2) | (A-2-a) | Biases against patients’ race or sexual behaviors | “Unequal HIV prevention pill puts minority men at risk! Black and Latino gay men are less likely to take Truvada as PrEP or largely because doctors don’t recommend it to them”—Reddit | 5 (38%) |
|  | Mistake (A-3) | (A-3-a) | The physician failed to renew patients’ prescription | “Went to go get my refill for Descovy at the pharmacy and found out that my doctor forgot to send my refill prescription. I ran out of pills in a few days. The pharmacist told me they couldn’t refill it without a doctor’s note. I’ve tried everything so fat since this is an emergency and nothing has worked. Now I’m here waiting.”—Reddit | 1 (8%) |
| **Patient level (B)** | | | | | 570 (72.61) |
|  | **Knowledge (B-1)** | | | | |
|  |  | (B-1-a) | Low awareness of PrEP and low demand for PrEP | “It’s upsetting to know people who have no idea that Truvada and other generic brands exist. HIV negative people can take this as a prevention and yes it has side effects, but it’s an amazing option for many people”—Tumblr | 11 (1.9%) |
|  |  | (B-1-b) | Sharing PrEP knowledge or experience with other patients | “Went to the clinic for a follow up after completing PrEP. After advice and a reassuring kind hand, my health advisor put me back on Truvada and Isentress. Both my HA and Doctor made me feel much calmer about my chances”—Tumblr (incorrect information, as this combination therapy is not approved for PrEP but instead for HIV treatment and postexposure prophylaxis). “Last year I bought Truvada for PrEP and I chose on demand dosing (2 pills the day before then 1 pill every day ending two days after last encounter). I think it cost me around 1000 GBP”—Reddit | 164 (28.8%) |
|  |  | (B-1-c) | Information on taking PrEP to prevent COVID-19 (asking or sharing) | “Hey Lopimune has shown to be effective against coronavirus, but no one mentions other HIV meds. Tenofovir is also cheap! Could any pharmacist who has knowledge of how things work tell me how effective it is against coronavirus?”—Reddit | 13 (2.3%) |
|  |  | (B-1-d) | Asking about knowledge related to the use, effectiveness, or side effect of PrEP | “If you have any bone loss (osteopenia), do you qualify for Descovy?”—Twitter | 113 (19.8%) |
|  |  | (B-1-e) | Comparing different drugs (Truvada and Descovy) | “They switched me from Truvada to Descovy. The last test you sent is the title of your autobiography”—Twitter | 263 (46.1%) |
|  |  | (B-1-f) | Asking about insurance coverage on PrEP (changing coverage or information sharing) | “Health insurance that covers the prescription is what I needed. HIV pill is $3k a month. The ACA doesn’t cover tier 1 drugs”—Twitter | 20 (3.5%) |
|  |  | (B-1-g) | COVID-19 for people on PrEP | “On Descovy for PrEP. I had 103 fevers, chills and was exhausted. Which sounds like symptoms of COVID. I heard someone recently recovered from COVID. I’m wondering if I had the virus or not”—Reddit | 9 (1.6%) |
|  |  | (B-1-h) | Asking how to donate their PrEP drug | “I recently switched to Descovy and am looking to get rid of 3 unopen bottles of Truvada. I hate to see someone waste thousands of dollars of medication when I can just give them mine”—Reddit | 4 (0.7%) |
|  | **Attitudes and beliefs (B-2)** | | | | |
|  |  | (B-2-a) | Side effects, effectiveness, toxicities, and interaction with feminizing hormones | “Hey friends. I went to my primary to get lab work done and was approved for PrEP. I have the choice between Truvada or Descovy since they are both covered. I’m concerned of weight gain by Descovy but I think that’s for treating people with HIV not PrEP. Can anyone help me out? My doctor was unsure”—Reddit | 82 (14.4%) |
|  |  | (B-2-b) | Managing multiple health concerns and PrEP side effects | “I have HSV2 and since I get frequent outbreaks, I moved from a standard 3 days valyclovir to daily suppressive therapy. I take 1 pill daily for PrEP. Is it a bad idea to take two medications daily?”—Reddit | 5 (0.9%) |
|  |  | (B-2-c) | Prioritization of care for current conditions (eg, pain or stress) above HIV prevention | “I’ve been looking into PrEP for some time now and I’m ready to go to a clinic to get some, but I found something that concerns me. I take medication called Tegretol as a mood stabilizer for bipolar. I’ve been on it for years and I don’t want to go off it. However, it’s one of those drugs that interacts with a lot of others. Since Truvada is a fixed dose pill, can I assume I don’t really have another option. Has anyone dealt with this specific problem before or are their any medical professionals that can give me some guidance?”—Reddit | 1 (0.2%) |
|  |  | (B-2-e) | Distrust of medical system: structural racism, transphobia, and negative experiences | “In my inner circle, I’ve noticed that my friends express a lot of medical mistrust when it comes to their daily checkups or any medical related subject. I’m not sure if it’s because of their insurance or if it’s because of how they were brought up. But everytime the subject is brought up they express a lot of negative experiences they’ve been through. I hope it gets better for them””—Reddit | 1 (0.2%) |
|  |  | (B-2-f) | Competing priorities during periods of substance use | “Happy New Year! I’m a long-time substance user; specifically, LSD and mushrooms. I recently tried Molly at a concert and WOW! It’s something I look forward to doing again. I’m also interested in conduct experiments with multiple drugs. I’ve searched everywhere on reddit and have found not post related to taking Molly with PrEP medication. I’m on Descovy daily to prevent HIV exposure. Does anyone know of contraindications with recreational drugs? I’ve heard that it could be bad on the kidneys, so I’d prefer to avoid similar side effects. Please, message me if you can answer my question”—Reddit | 2 (0.4%) |
|  |  | (B-2-g) | Diminished concern for prevention with intimate partners | “Been on PrEP for a few years now so I can protect myself from HIV. But now I’m in a stable relationship with a man I trust, and I think it’s time that I put the pill down once I run out in two weeks. I just have no more use for it. My mind is made up, but I do want to hear if any of you fine gents have also quit PrEP”—Reddit | 2 (0.4%) |
|  |  | (B-2-i) | Unwillingness to discuss PrEP with primary care providers | “I’m waiting to obtain PREP affordably through the government’s funded scheme, but I have to get a prescription. I don’t want to go to a clinic I’m actually enrolled in for personal reasons. Does anyone here know where I can book an appointment without having all my files on record?”—Reddit | 1 (0.2%) |
|  |  | (B-2-j) | PrEP vs another protection approach (use of a condom) | “It’s always advisable to use a condom if you’re not covering both sides. I’m on both PrEP and STI/UTI injection, it’s good to be safe”—Twitter | 50 (8.8%) |
|  |  | (B-2-k) | Self-evaluated as low risk | “I’ve been on PrEP for 7 months now. Since the pandemic started I’m wondering if I should take half a pill or take every 2 days since I’m not getting much interaction because of the lockdowns and social distancing”—Reddit | 10 (1.8%) |
|  |  | (B-2-l) | Concerns about the privacy of health information (under same coverage with family) | “Hello I’m 23 and live with my mother. I want to get PrEP but one thing I’m worried about is her judgement and don’t know how to conceal the drug or checkups every few months. Any advice?”—Reddit | 10 (1.8%) |
| **Community level (C)** | | | | | 166 (21.15) |
|  | Communication and awareness (C-1) | (C-1-a) | Lack of effective messaging about PrEP | “I don’t understand why there isn’t more around a PrEP vaccine. I mean when I saw posters up around my city, I could hardly believe it. It’s not a cure, but a huge wave in fighting HIV. Prevention is important to stopping it’s spread but it wasn’t on the news or at least where I live. A lot of people around me don’t know a thing. Shouldn’t I be more ecstatic than this? Shouldn’t we remind students everywhere to get their shots? We should be spreading the word!”—Tumblr | 5 (3%) |
|  | **Funding (C-2)** | | | | |
|  |  | (C-2-a) | Limited health budgets to sustain PrEP program | “With insurance, Descovy costs me 80 a month. Does anyone have advice on lowering the cost?”—Reddit | 55 (33.1%) |
|  |  | (C-2-b) | Lack of insurance coverage and financial assistance programs ~~Capacity and access~~ | “My insurance forced me to use the generic version for Truvada and now I can’t use my Gilead coupon”—Twitter | 35 (21.1%) |
|  | **Capacity and access (C-3)** | | | | |
|  |  | (C-3-a) | Lack of focus on *nonprescribing service providers* | “I heard you can get Truvada available for 30-60 days without a doctor’s prescription. Why can’t Truvada be a non-prescription medicine then?””—Twitter | 1 (0.6%) |
|  |  | (C-3-c) | Lack of training, referral systems, or established reimbursement levels for care and drugs | “Why is getting Truvada a hassle? It took me 3 months to get a prescription and now I’m having to jump through a number of hoops to get it filled”—Tumblr | 3 (1.8%) |
|  |  | (C-3 d) | Legal constraints to providing PrEP for youth, including mandates to involve parental figures in working with minors | “I’m 17 years old living in California and recently I’ve been taking this online sex-ed program called SMART. It focuses on HIV prevention and I’m confident that I want to and should take PrEP. I’ve been looking at resources for CA teens and so far, I’m really confused. Currently, I face two problems. I’m not out to my parents so I don’t want them finding out (maybe from insurance bills) If anyone has any ideas on getting PREP as a teen or from a country confidentially over time if need be, I’d appreciate the advice”—Reddit | 8 (4.8%) |
|  |  | (C-3-e) | Lack of access to care: inadequate transportation, inflexible work schedules, and inconvenient locations dispensing PrEP | “I’m having trouble deciding which one to use. Just moved to a different city and my doctor who is 4 hours away is kinda far. My major issue is I can’t find in-network doctors near me that actually prescribe it”—Reddit | 16 (9.6%) |
|  |  | (C-3-f) | Time constraints on medical appointments | “Why can’t HIV related help be done through telemedicine? Especially during the age of COVID. If we can take classes online through Zoom why can’t we do doctor appointments for HIV online also? I wouldn’t mind talking to a doctor then going to a facility to do blood work if necessary””—Reddit | 2 (1.2%) |
|  |  | (C-3 g) | Lack of medical insurance and limited insurance networks | “Go to Callen-Lorde Community Health Center, they offer free healthcare for LGBTQ :). It’s funded by donations and I got a free PrEP physicals testing etc from there”—Twitter | 9 (5.4%) |
|  |  | (C-3 h) | Lack of patient confidence and perseverance to access care | “One of the fragile parts of Truvada is there’s no lifelong tests about its side effects. Truvada hasn’t been out as long as *aspirin*”—Reddit | 4 (2.4%) |
|  |  | (C-3-i) | Political event (VOTE for “PrEP should be available for everyone”) | “(Inline Graphic 1) Having sex with someone who is living with HIV and undetectable is safe sex. It cannot be passed on. #UequalsU (Inline Graphic 1) #Condoms are important for preventing STIs and unwanted pregnancy. (Inline Graphic 1) #PrEP must be available to anyone who wants it. (Inline Graphic 1) #Voting is one of the most impactful things you can do for yourself and your community. #WellnessWednesday #AskUsAboutPrEP #PrEP #BeTheGeneration”—Instagram | 2 (1.2%) |
|  | Pharmaceutical barriers (C-4) | (C-4-a) | Constraints of Truvada as PrEP (eg, daily dosing schedule and side effects) | “I came off PREP following a high risk unprotected receptive sex. I was put on Truvada and Isentress. For me to have stress of adhering to pills made me surprised. I’m happy to live stress free without taking pills, but 4 days later I’m back into the position of where I need to get PrEP again. I just don’t understand what my life is anymore”—Tumblr | 21 (12.7%) |
|  | **Population-specific barriers and stigma (C-5)** | | | | |
|  |  | (C-5-a) | Lack of transinclusive marketing of PrEP | “The DISCOVER trial conducted to get its FDA approval only included cisgender men and transgender women who have had sex with males. Maybe it’s smart to be studying cis females also”—Reddit | 28 (16.9%) |
|  |  | (C-5-b) | Low prioritization of PrEP for people who inject drugs | “@apoorva_nyc Fantastic! What’s also urgently needed for LAI cabotegravir testing is #HIV transmission among injection drug users. 1 in 10 HIV transmission happens among IDUs or IDU+ MSM. PrEP can do wonders for this vulnerable population if tested and made accessible”—Twitter | 2 (1.2%) |
|  |  | (C-5-c) | The stigma associated with PrEP use and accessing HIV services | “I’m a gay man and on PrEP. I take Truvada every day since I hook up with a lot of guys. Truvada basically makes me immune from getting HIV through sex. Should I tell my recruiter that it’s the only med I take?”—Reddit | 18 (10.8%) |
|  |  | (C-5 d) | The intersection of HIV stigma with transphobia and homophobia | “Stop being naive. PrEP does not prevent any other STI. The amount of raw sex in the gay community is a problem. I’ve seen first-hand how it affects people’s lives”—Twitter | 4 (2.4%) |
|  |  | (C-5-e) | Lack of cis women–inclusive drug trials | “Today I learned that one of the PrEP drugs was tested on cis men and trans women because they didn’t test it on cis women or trans men. It got approved for use for those groups. They didn’t think it would be effective, so they didn’t bother trying” ”—Reddit | 2 (1.2%) |

^a^PrEP: pre-exposure prophylaxis.
