# Supplementary material for: Unsupervised Machine Learning to Detect and Characterize Barriers to Pre-exposure Prophylaxis Therapy: Multiplatform Social Media Study
Source: JMIR Infodemiology. 2022 Apr 28;2(1):e35446. doi: 10.2196/35446 (PMC10014091; doi:10.2196/35446)
Supplement: Multimedia Appendix 2 [file infodemiology_v2i1e35446_app2.docx]

### Multimedia Appendix 2

#### Breakdown of Topics by Platform

**Instagram**

Among the total 41 signal posts on Instagram, 32 posts were under patient-level topics, 8 community level, and 1 provider level. Topics comparing PrEP and another protection approach (Code number: B-2-j) had the highest volume (16 posts), followed by sharing knowledge and personal experience with another patient (Code number, B-1-b) (8 posts); and 4 posts on a topic related to constraints of Truvada as PrEP (Code number, C-4-a.) (See table 4)

**Reddit**

Among the total 256 signal posts in Reddit, 102 posts were under patient-level topics, 71 community level, and 9 posts under the provider level. The top three topics with the most posts (78 posts) were for the topic inquiring about knowledge related to the use or effectiveness of PrEP (Code number: B-1-d), the second highest topic (77 posts) on Reddit was a topic reflecting on why patients were concerned about side effects; effectiveness; toxicities; and the interaction with feminizing hormones (Code number, B-2-a), followed posts (31 posts) with topics associated with a patient reporting limited health financial resources for sustaining PrEP therapy (Code number: C-2-a).

**Tumblr**

Similar to Instagram and Reddit, among the total 41 signal posts in Reddit, patient-level topics had the highest volume (29 posts), followed by the community level (9 posts), and 1 post under the provider level. The top three topics in Tumblr were: 1) sharing PrEP knowledge or experience with other patients (Code number: B-1-b) (35 posts); 2) topics (5 posts) associated with concerns about side effects; effectiveness; toxicities; and the interaction with feminizing hormones (Code number, B-2-a), and 3) topics (3 posts) about limited health financial resources for sustaining PrEP therapy (Code number: C-2-a).

**Twitter**

Twitter had the highest volume of signal posts among all selected social media platforms. On Twitter, patient-level topics also had the highest volume of posts (398 posts), followed by the community level (71) and the provider level (2). The top three topics on Twitter were all topics under the patient-level including conversations comparing Truvada and Descovy (242 posts) (Code number: B-1-e); topics sharing PrEP knowledge with other patients (111 posts) (Code number: B-1-b); and inquiring about the use, effectiveness, or side effect of PrEP (35 posts) (Code number: B-1-d).

**YouTube**

We were able to detect 17 signal posts on YouTube, including 8 patient-level signals, 8 community level signals, and 1 provider level signal. These signal posts covered 9 topics, a topic about the impact of COVID -19 on people using PrEP (Code number: B-1-g) had the highest volume (4 posts), followed by a topic related to the constraints of Truvada as PrEP therapy (Code number: C-4-a) (3 posts), and conversations comparing Truvada and Descovy (Code number: B-1-e) (2 posts).

Supplementary Table 2: Top 3 topics in selected five social media platforms.

| **Platform** | **Top 3 Topic Code number** | **Topic description** | **Number of Post** |
| --- | --- | --- | --- |
| **Instagram** | B-2-j | PrEP vs another protection approach (use of a condom) | 16 |
|  | B-1-b | Sharing PrEP knowledge or experience with other patients | 8 |
|  | C-4-a | Constraints of Truvada as PrEP (e.g., daily dosing schedule, side effects) | 4 |
| **Reddit** | B-1-d | Asking about knowledge related to the usage or effectiveness or side effect of PrEP | 78 |
|  | B-2-a | Side effects; effectiveness; toxicities; interaction with feminizing hormones | 77 |
|  | C-2-a | Limited health budgets on sustain PrEP program | 31 |
| **Tumblr** | B-1-b | Sharing PrEP knowledge or experience with other patients | 32 |
|  | B-2-a | Side effects; effectiveness; toxicities; interaction with feminizing hormones | 5 |
|  | C-2-a | Limited health budgets on sustain PrEP program | 3 |
| **Twitter** | B-1-e | Comparing different drugs (Truvada, Descovy) | 242 |
|  | B-1-b | Sharing PrEP knowledge or experience with other patients | 111 |
|  | B-1-d | Asking about knowledge related to the usage or effectiveness or side effect of PrEP | 35 |
| **YouTube** | B-1-g | COVID-19 for people on PrEP | 4 |
|  | C-4-a | Constraints of Truvada as PrEP (e.g., daily dosing schedule, side effects) | 3 |
|  | B-1-e | Comparing different drugs (Truvada, Descovy) | 2 |
